# Association of genetic polymorphisms in the *C19orf66* gene and biochemical indices of HBV infected individuals in Yunnan

**DOI:** 10.3389/fcimb.2023.1180366

**Published:** 2023-05-24

**Authors:** Ni Liu, Min Liu, Jun Yang, Shuwei Dong, Ming Yue, Peng Huang, Xueshan Xia, A-Mei Zhang

**Affiliations:** ^1^ Faculty of Life Science and Technology, Kunming University of Science and Technology, Kunming, Yunnan, China; ^2^ Department of Infectious Disease and Hepatic Disease, First People’s Hospital of Yunnan Province, Kunming, Yunnan, China; ^3^ Clinical Laboratory, The People’s Hospital of Maguan County, Wenshan, Yunnan, China; ^4^ School of Public Health, Nanjing Medical University, Nanjing, China; ^5^ Kunming Medical University, Kunming, Yunnan, China

**Keywords:** HBV infection, the *C19orf66* gene, genetic polymorphisms, biochemical indices, functional assay

## Abstract

**Introduction:**

Hepatitis B virus (HBV) infection causes serious liver diseases and is a healthy problem worldwide. Although vaccines are administered to infants after birth, there is no effective medicine for HBV infection. The interferon-stimulated genes (ISGs) are important factors in the host that can aid in restraining the virus, and the *C19orf66* gene has a wide-antiviral spectrum.

**Methods:**

In this study, three SNPs in the *C19orf66* gene were sequenced and genotyped, and their potential function were predicted and further verified by dual-luciferase reporter assay.

**Results:**

Although no significant difference of genotype and allele frequency was observed between HBV patients and the controls, the genotype and allele frequency showed significant difference between HBV patients with HBsAg-positive and HBV patients with HBsAg-negative or controls. Genotype AA (*P*= 0.009) and AT (*P*= 0.019) of rs77076061 showed higher and lower frequency in HBV patients with HBsAg-positive than in patients with HBsAg-negative, respectively. Genotype AG of rs1979262 played a risk role in HBV patients with HBsAg-positive (13.22%) than in patients with HBsAg-negative (7.53%, *P*= 0.036) or controls (8.48%, *P*= 0.033). The frequency of allele A of rs1979262 was higher in patients with HBsAg-positive (6.61%) than in patients with HBsAg-negative (3.77%, *P*= 0.042), while it was the opposite for the allele G. Moreover, the associations between genotypes of SNPs in the *C19orf66* gene and the ALT, AST, and DBIL level were also identified. The functional assay suggested that the SNPs might influence the *C19orf66* expression by changing the connection of transcriptional factors.

**Conclusion:**

In summary, the association between genetic polymorphisms in the *C19orf66* gene and HBV infection/biochemical indices of patients was firstly identified in Yunnan Province.

## Introduction

During 2020 and 2021, the highest cause of morbidity of all infectious diseases in China is accredited to viral hepatic disease (Sarin et al., 2020), and most infectious hepatic viruses were hepatitis B virus (HBV) and hepatitis C virus (HCV). Until now, there has been no effective cure for HBV-infected individuals. Thus, studying the infectious progress and pathogenic mechanisms of HBV infection is necessary.

Host immunology is the first-line of defense against viral infection, and several genetic polymorphisms of the immune genes have been identified to be associated with HBV infection (Zeng et al., 2021; Chen et al., 2022). Interferons (IFNs) and interferon-stimulated genes (ISGs) induced by IFNs are important anti-viral factors of the host. Genetic polymorphisms in the *IL28B*, *IFNL4*, *MxA*, and *MxB* gene were identified to be associated with HBV infection in the Yunnan population (Zhang et al., 2014; Zheng et al., 2022). These results suggested the necessary roles of IFNs and ISGs in HBV infection. In 2011, the *C19orf66* gene was reported as an interferon-stimulated gene for the first time. Then, the anti-virus effects of the *C19orf66* gene were identified against human immunodeficiency virus (HIV), HCV, Kaposi’s sarcoma-associated herpesvirus (KSHV), and dengue virus (DENV) (Suzuki et al., 2016; Rodriguez et al., 2019; Wang et al., 2019; Kinast et al., 2020). Although the anti-viral mechanisms were different for the various infection, the results suggested a wide-spectrum of anti-viral factor. There has been no study to analyze the role of the *C19orf66* gene in HBV infection.

This is the first study to analyze the genetic and functional roles of the *C19orf66* gene in HBV infection and disease progression.

## Materials and methods

### Individuals

A total of 655 HBV-infected individuals and 448 normal controls were recruited by the doctors of the First people’s hospital of Yunnan Province and the people’s hospital of Maguan in this study. HBV infection were confirmed by assessing the five serological HBV indices and clinical symptoms by the doctors. HBV patients and controls were detected as HCV or HIV non-infection, and the controls were further identified without any other infectious disease and liver disease. All HBV patients were recruited as chronic HBV infection, and samples were collected before treatment. The basic information and biochemical indices, including liver function test and renal function test, were collected for analysis, and 3 mL of whole blood was obtained from each individual. The biochemical indices include the alanine transaminase (ALT), aspartate transaminase (AST), total bilirubin (TBIL), direct bilirubin (DBIL), indirect bilirubin (IBIL), total protein (TP), albumin (ALB), globin (GLOB), blood urea nitrogen (BUN), serum creatinine (CREA), and serum uric acid (UA). Written informed consent was obtained from each participant, conforming to the tenets of the Declaration of Helsinki before the study. The Institutional Review Board of Kunming University of Science and Technology approved the present study.

### Genomic DNA extraction and SNP genotyping

Genomic DNA was extracted from 200 μL whole blood sample of each patient by using TIANamp Blood DNA Kit (TIANGEN, China). Three single nucleotide polymorphisms (SNPs, rs77076061, rs1979262, and rs12611087) were selected according to the criteria that the minor allele frequency of SNP is more than 2% in dbSNP (www.ncbi.nlm.nih.gov/SNP). these SNPs were considered as tag SNPs in NCBI database (ftp.ncbi.nlm.nih.gov/hapmap/, CHB + JPT). SnapShot method, which is a genotyping method based on the single-base extension with fluorescence, was used to genotype these SNPs. Three SNPs of some samples were sequenced and genotyped using the Sanger sequencing method to verify the results of SnapShot (Accession Numbers: OQ828718 - OQ829276). The amplification and sequencing primers are listed in **Supplementary Table 1**.

### *In silico* prediction of three SNPs of the *C19orf66* gene

All three SNPs were located in the intron region of the *C19orf66* gene. The HaploReg v4.1 website was used to annotate and analyze the function of each SNP. Then, the annotation of each SNP was explained according to Roadmap Epigenomics Consortium (Roadmap Epigenomics et al., 2015). Finally, the potential function of each SNP was predicted.

### Cell culture and plasmids construction

The HepG2 cells were cultured in Dulbecco’s modified Eagle medium (DMEM, Hyclone, USA) together with 10% fetal bovine serum (FBS), 1 mM pyruvate, 2 mM L-glutamine, 100 μg/mL streptomycin and 100 U/mL penicillin at 37°C with 5% CO_2_ incubator.

To verify the function of SNPs in this study, DNA regions containing two alleles of each SNP, which were only located on the introns of the *C19orf66* gene (rs77076061 locates on intron 1, rs1979262 and rs12611087 locate on intron 3), were amplified. The restriction endonuclease sites were designed in the 5′-end of each primer (*Kpn* I site in forward primers and *Hind* III site in reverse primers are underlined in **Supplementary Table 2**). The PCR products were then constructed into the pGL3-basic plasmid by using the restriction endonuclease method.

### Transfection and dual-luciferase reporter assay

Cells were cultured and transfected by using the mixture of 4.2 μg plasmids (including 4 μg firefly luciferase pGL3-basic plasmids and 0.2 μg renilla luciferase pRL-SV40 plasmids) and 3 μL Lipofectamine 3000 (Life, USA) in 12-well plate. Then, the cells were harvest at 24, 48, and 72 h for further studies. Dual-luciferase reporter assays (Beyotime, China) were performed to evaluate the function of two allele of each SNP. The intensities of firefly luciferase and Renilla luciferase (used as controls) were detected at 560 and 465 nm by using Victor X Multimode Plate Reader (PerkinElmer, USA), respectively. Finally, the firefly/Renilla ratio was calculated to analyze the expression levels of firefly.

### Data analysis

The genotype and allele frequencies of each SNP were analyzed among the HBV patients with HBsAg-positive, the patients with HBsAg-negative, and the normal controls by using the Chi-square test with Yates’ correction or Fisher’s exact test. The biochemical indices were represented as mean ± SEM in HBV patients and controls. The differences of biochemical indices were analyzed by using Student’s t-test (two-tailed) among HBV patients with different genotypes of each SNP. A *P*-value less than 0.05 was considered to be statistically significant.

## Results

### Genotype and allele frequencies analysis

In total, HBV patients included 345 males and 310 females in, and there were 275 males and 173 females in the controls. The males occupied 52.67% and 56.35% of total samples in HBV patients and controls, respectively. The mean age of controls and HBV patients was 40.58 ± 0.53 and 40.82 ± 0.38, respectively. No significant difference was observed in the genotype and allele frequencies of three SNPs between HBV infected persons and controls (**Table 1**, **Supplementary Table 3**).

**Table 1:** Analysis of genotypes and alleles in the C19orf66 gene between HBV patients and normal controls.

SNP	HBV patients (N= 655)	Controls (N=448)	*P*-value	OR (95% CI)
rs77076061				
Genotype AA	11	4	0.399	1.896 (0.592-5.461)
AT	104	66	0.665	1.092 (0.787-1.523)
TT	540	378	0.446	0.870 (0.627-1.200)
Allele A	126	74	0.309	1.182 (0.878-1.606)
T	1184	822		0.846 (0.623-1.139)
rs1979262				
Genotype AA	0	3	0.131	0 (0-0.788)
AG	73	38	0.180	1.353 (0.906-2.020)
GG	582	407	0.333	0.803 (0.537-1.206)
Allele A	73	44	0.559	1.143 (0.774-1.682)
G	1237	852		0.875 (0.595-1.292)
rs12611087				
Genotype CC	502	347	0.808	0.955 (0.720-1.268)
CT	142	92	0.703	1.071 (0.801-1.431)
TT	11	9	0.459	0.643 (0.277-1.600)
Allele C	1146	786	0.917	0.978 (0.756-1.263)
T	164	110		1.023 (0.792-1.323)

The HBsAg was a bio-marker for HBV cccDNA replication in the clinic, and therefore, the HBV patients were divided into HBsAg-positive and -negative for further analysis. The results showed a statistical difference between HBV patients with HBsAg-positive and patients with HBsAg-negative or controls (**Table 2**). Genotype AA of rs77076061 showed higher frequency in HCV patients with HBsAg-positive (2.64%) than in patients with HBsAg-negative (0%, *P*= 0.009). The frequency of genotype AT of rs77076061 was lower in patients with HBsAg-positive (13.22%) than in patients with HBsAg-negative (20.50%, *P*= 0.019). Genotype AG of rs1979262 showed significantly higher frequency in HBV patients with HBsAg-positive (13.22%) than in patients with HBsAg-negative (7.53%, *P*= 0.036) or controls (8.48%, *P*= 0.033). Moreover, the frequency of genotype GG of rs1979262 was 86.78% and 92.47% in patients with HBsAg-positive and HBsAg-negative, respectively, and a significant difference was observed. The frequency of allele A of rs1979262 was higher in patients with HBsAg-positive (6.61%) than in patients with HBsAg-negative (3.77%, *P*= 0.042), while it was opposite for the allele G. These results suggested the protective roles played by genotype AT of rs77076061 and genotype GG of rs1979262 in HBV patients with HBsAg-positive.

**Table 2:** Analysis of genotypes and alleles in the C19orf66 gene between HBV patients with HBsAg-positive and HBsAg-negative.

SNP	HBV patients with HBsAg-positive (N= 416, group #1)	HBV patients with HBsAg-negative (N= 239, group #2)	Controls (N=448, group #3)	#1 vs. #2 *P*-value (OR, 95%CI)	#1 vs. #3 *P*-value (OR, 95%CI)	#2 vs. #3 *P*-value (OR, 95%CI)
rs77076061						
Genotype AA	11	0	4	0.009 (0.0001, 0.00-0.621)	0.067 (3.015, 0.938-8.688)	0.304 (0, 0-1.880)
AT	55	49	66	0.019 (0.591, 0.388-0.906)	0.557 (0.882, 0.606-1.296)	0.068 (1.493, 0.999-2.243)
TT	350	190	378	0.163 (1.368, 0.913-2.060)	0.926 (0.982, 0.680-1.423)	0.133 (0.718, 0.482-1.066)
Allele A	77	49	74	0.623 (0.893,0.611-1.291)	0.496 (1.133, 0.810-1.588)	0.257 (1.269, 0.875-1.863)
T	755	429	822	0.623 (1.120, 0.775-1.636)	0.496 (0.883, 0.630-1.235)	0.257 (0.788, 0.537-1.142)
rs1979262						
Genotype AA	0	0	3	–	0.250 (0, 0-1.241)	0.555 (0, 0-2.142)
AG	55	18	38	0.036 (1.871, 1.081-3.275)	0.033 (1.644, 1.061-2.560)	0.774 (0.879, 0.481-1.569)
GG	361	221	407	0.036 (0.535, 0.305-0.925)	0.073 (0.661, 0.434-1.011)	0.563 (1.237, 0.704-2.237)
Allele A	55	18	44	0.042(1.809, 1.044-3.095)	0.157 (1.371, 0.911-2.052)	0.402 (0.758, 0.432-1.324)
G	777	460	852	0.042(0.553, 0.323-0.958)	0.157 (0.730, 0.487-1.098)	0.402 (1.320, 0.755-2.314)
rs12611087						
Genotype CC	311	191	347	0.160 (0.744, 0.505-1.102)	0.396 (0.862, 0.634-1.172)	0.517 (1.158, 0.782-1.707)
CT	99	43	92	0.102 (1.424, 0.956-2.116)	0.283 (1.208, 0.875-1.674)	0.485 (0.849, 0.569-1.270)
TT	6	5	9	0.759 (0.685, 0.201-1.982)	0.707 (0.714, 0.255-1.890)	0.834 (1.042, 0.387-2.982)
Allele C	721	425	786	0.271 (0.810, 0.570-1.147)	0.555 (0.909, 0.686-1.205)	0.574 (1.122, 0.793-1.595)
T	111	53	110	0.271 (1.235, 0.872-1.755)	0.555 (1.100, 0.830-1.458)	0.574 (0.891, 0.627-1.262)

Group #1 means HBV patients with HBsAg-positvie; Group #2 means HBV patients with HBsAg-negative; Group #3 means general controls; P-value less than 0.05 is statistically significant.

### Biochemical indices showed significant difference among patients with different genotypes

After studying the biochemical indices among HBV patients with different genotypes of each SNP, statistical differences were observed (**Table 3**). The ALT, AST, and DBIL levels showed significant difference among patients with different genotypes of rs77076061 and rs12611087. The AST and ALT level were significantly higher in HBV patients with genotype TT of rs77076061, and the DBIL level was lower in patients with genotype AA of rs77076061. Patients with genotype TT of rs77076061 might prefer to serious liver disease. The patients with genotype TT of rs12611087 showed lower AST, ALT, DBIL, and CREA levels than that in other patients. Genotype TT of rs12611087 might play a protective role in disease progression of HBV infection. The TP level was statistically higher in patients with genotype AG of rs1979262 than that of other patients. These results showed that genetic polymorphisms of the *C19orf66* gene were associated with biochemical indices of HBV patients in Yunnan.

**Table 3:** Biochemical indices analysis among HBV patients with various genotypes. (Welch’s ANOVA test, and unpaired t test with Welch’s correction).

Biochemical features (reference range)	rs77076061				rs1979262		
	AA	AT	TT	*P*-value	AG	GG	*P*-value
AST (5-40 U/L)	29.59 ± 6.87	32.35 ± 2.67	46.31 ± 4.64	0.038	38.08 ± 5.30	44.57 ± 4.30	0.343
ALT (5-40 U/L)	30.03 ± 4.96	37.93 ± 4.81	53.78 ± 6.80	0.025	42.65 ± 5.45	52.03 ± 6.37	0.264
TBIL (3.4-20.5 µmol/L)	13.14 ± 1.87	17.42 ± 3.10	17.80 ± 1.20	0.123	19.00 ± 3.55	17.50 ± 1.16	0.690
DBIL (0-6.8 µmol/L)	4.29 ± 0.52	7.54 ± 2.38	7.76 ± 0.91	0.003	7.76 ± 2.44	7.66 ± 0.90	0.968
IBIL (0-13.7 µmol/L)	8.85 ± 1.51	9.88 ± 0.83	10.04 ± 0.35	0.747	11.23 ± 1.21	9.84 ± 0.32	0.268
TP (65-80 g/L)	73.72 ± 2.41	73.01 ± 0.78	73.39 ± 0.31	0.894	74.98 ± 0.80	73.13 ± 0.31	0.034
ALB (35-55 g/L)	42.33 ± 1.81	40.52 ± 0.61	41.48 ± 0.26	0.332	42.37 ± 0.62	41.21 ± 0.26	0.087
GOLB (20-30 g/L)	31.39 ± 1.29	32.49 ± 0.56	31.83 ± 0.24	0.528	32.54 ± 0.77	31.85 ± 0.23	0.393
BUN (2.0-7.1 mmol/L)	7.13 ± 2.99	5.16 ± 0.55	4.86 ± 0.11	0.668	5.18 ± 0.81	4.92 ± 0.12	0.751
CREA (45-84 µmol/L)	114.8 ± 54.73	73.82 ± 9.22	72.95 ± 3.65	0.757	64.12 ± 2.10	74.72 ± 3.80	0.015
UA (178-416 µmol/L)	391.3 ± 40.12	337.5 ± 10.36	357.2 ± 16.61	0.335	330.0 ± 12.36	357.2 ± 15.26	0.168
Biochemical features (unit)	rs12611087						
	CC	CT	TT	*P*-value			
AST (5-40 U/L)	43.87 ± 4.72	45.15 ± 6.42	24.01 ± 3.27	0.0005			
ALT (5-40 U/L)	51.54 ± 7.17	51.00 ± 7.17	23.93 ± 4.26	0.0004			
TBIL (3.4-20.5 µmol/L)	16.86 ± 1.05	20.77 ± 3.42	12.77 ± 2.08	0.107			
DBIL (0-6.8 µmol/L)	7.13 ± 0.81	9.76 ± 2.57	4.30 ± 0.88	0.026			
IBIL (0-13.7 µmol/L)	9.74 ± 0.31	11.01 ± 0.94	8.47 ± 1.33	0.289			
TP (65-80 g/L)	73.17 ± 0.33	73.91 ± 0.59	72.91 ± 2.42	0.559			
ALB (35-55 g/L)	41.23 ± 0.27	41.70 ± 0.53	41.38 ± 1.16	0.739			
GOLB (20-30 g/L)	31.86 ± 0.25	32.19 ± 0.42	31.58 ± 2.18	0.796			
BUN (2.0-7.1 mmol/L)	4.97 ± 0.16	4.91 ± 0.20	3.91 ± 0.41	0.073			
CREA (45-84 µmol/L)	75.00 ± 4.24	70.14 ± 4.84	58.04 ± 4.75	0.035			
UA (178-416 µmol/L)	344.3 ± 5.15	392.3 ± 64.74	369.1 ± 31.75	0.588			

The mean ± SEM of each biochemical indice among HBV patients with variant genotypes was analyzed by using Student’s t-test (two-tailed).

### *In silico* prediction

According to the prediction of HaploReg v4.1, three SNPs regions play regulatory roles in expression of the *C19orf66* gene. Based on the Roadmap Epigenomics Consortium, all three SNPs were located on the peak of H3K4me1, H3K4me3, and H3K27ac, which are considered as the promoter or enhancer regions and regulates the progress of gene transcription.

### The function assay of three SNPs

**Figure 1:**
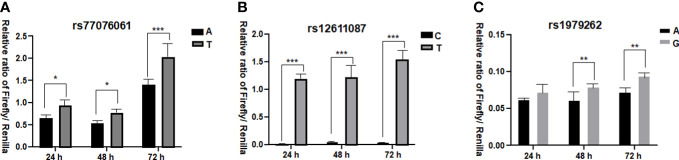
Dual-luciferase reporter assay. **(A)** the relative ratio of Firefly/Renilla for allele A and T of rs77076061; **(B)** the relative ratio of Firefly/Renilla for allele C and T of rs12611087; **(C)** the relative ratio of Firefly/Renilla for allele A and G of rs1979262. * means *P*< 0.05, ** means *P*<0.01, *** means *P*< 0.001.

The dual-luciferase reporter assay was performed to verify the functional difference between two alleles of each SNP (**Figure 1**). The results showed that allele A of rs77076061 could significantly decrease firefly luciferase expression compared to allele T (**Figure 1A**). This result was consistent with the genetic association result, in which the genotype AA of rs77076061 showed significantly higher frequency in patients with HBsAg-positive (2.64%). The allele C of rs12611087 significantly decreased the firefly luciferase expression levels when compared with the plasmid transfected with allele T (**Figure 1B**). Although no significant difference was identified between rs12611087 and HBV infection or HBsAg-positive, the frequency of genotype CC of this SNP was a little lower in patients with HBsAg-positive (74.76%) than that in controls (77.46%) or in patients with HBsAg-negative (79.92%). In consonance with the genetic analysis, plasmids with allele A of rs1979262 showed significantly decreased firefly luciferase level than that of plasmids with allele G (**Figure 1C**). These results suggested that the possible function of genetic polymorphisms on disease progression through the change of location construction.

## Discussion

Interferon, ribavirin, and even the direct acting antiviral regiments seem ineffective in treating chronic HBV infection. There have been high rates of mortality each year due to serious liver diseases (hepatocirrhosis and hepatocellular carcinoma) caused by HBV infection (W.H.O, 2017). Studying the underlying mechanisms of HBV infection and the immune response of the host will help in developing viable therapeutic strategies. Many genetic polymorphisms of immune genes have been associated with HBV infection, disease progression, and treatment efficacy in HBV patients (Wei et al., 2021; Zheng et al., 2022).

ISGs are the main anti-viral factors of the host and also causative agent in treatment of HBV patients (Li et al., 2022). Genetic polymorphisms of the ISG genes have been associated with HBV infection, disease progression, and outcome in HBV patients. The SNPs in the *MxA* gene (King et al., 2002; Zheng et al., 2022) and the *MxB* gene were associated with biochemical indices and the response to interferon in HBV patients (Wang et al., 2020). The *C19orf66* gene, a novel ISG, has the anti-virus effects against both RNA virus and DNA virus. Although the anti-viral mechanisms of *C19orf66* were not similar, it was considered as a non-negligible and important factor for the host. Thus, it is essential to study the *C19orf66* gene in more details.

The *C19orf66* gene was identified to be a wide-spectrum anti-viral factor, but its role in HBV infection was not clear. Although many viruses could be inhibited by the *C19orf66* gene (Suzuki et al., 2016; Rodriguez et al., 2019; Wang et al., 2019), no genetic association study was performed. In this study, the genetic polymorphisms of the *C19orf66* gene in the HBV patients from Yunnan Province were analyzed. Although no association was found between genetic polymorphisms and HBV infection, the biochemical indices could be influenced by the genotypes of this gene. This result was similar to our previous study (Song et al., 2017; Song et al., 2021), in which the host genetic polymorphisms were associated with disease progression of HBV patients in Yunnan.

HBsAg is a biomarker to evaluate the replication of HBV cccDNA (Kramvis et al., 2022). Thus, the HBV patients were divided into groups with HBsAg-positive and HBsAg-negative for further analysis. The results revealed that the genotypes and alleles in the *C19orf66* gene were associated with the expression of HBsAg and further prompted HBV replication levels in the patients. Recently, Hepatitis B core-related antigen (HBcrAg), which included HBcAg, HBeAg, and P22cr protein, has been considered as a serum biomarker for the management of intrahepatic viral replicative activity (Inoue and Tanaka, 2020), suggesting its potential role as on-treatment monitoring marker for HBV patients in the clinic. However, all patients in this study were not treated during sample collection, and the relationship between SNPs in the *C19orf66* gene and the outcome could not be analyzed. Collecting samples from interferon-treated HBV patients may be considered in future investigations.

Although genotype and allele frequencies of three SNPs were similar between HBV patients and controls, allele A of rs1979262 was higher in HBV patients with HBsAg-positive than that of patients with HBsAg-negative. The dual-luciferase reporter assay was commonly used to investigate the potential function of SNPs in promoter region or intron (Chen et al., 2015; Mohebian et al., 2022). The allele A of rs1979262 significantly decrease the firefly expression level than allele G. This result was consistent with the genetic analysis. Thus, it suggested that allele A of rs1979262 might decrease expression level of the *C19orf66* gene by influence the combination of transcription factors. However, the effect mechanisms of the other two SNPs in this study need further investigated.

In conclusion, the association between genetic polymorphisms in the *C19orf66* gene and HBV patients with HBsAg/or biochemical indices of HBV patients was first identified by influencing the connection between this gene and transcription factors. The results suggested that the *C19orf66* gene might influence HBV replication and disease progression of HBV patients.

## Data availability statement

The datasets presented in this study can be found in online repositories. The names of the repository/repositories and accession number(s) can be found below: NCBI GenBank, OQ828718 - OQ829276.

## Ethics statement

The Institutional Review Board of Kunming University of Science and Technology approved the present study. The patients/participants provided their written informed consent to participate in this study.

## Author contributions

A-MZ and XX designed this study; NL and ML performed the experiments; JY, PH, and MY collected the samples; A-MZ and SD analyzed the data; A-MZ and NL prepared the manuscript. All authors contributed to the article and approved the submitted version.

## Supplementary material

Supplementary Table 3 Genotypes of each SNP in HBV and control samples.
