# Supplementary material for: Association of genetic polymorphisms in the C19orf66 gene and biochemical indices of HBV infected individuals in Yunnan
Source: Front Cell Infect Microbiol. 2023 May 24;13:1180366. doi: 10.3389/fcimb.2023.1180366 (PMC10245551; doi:10.3389/fcimb.2023.1180366)
Supplement: Supplementary file 1 [file Table_1.docx]

**Table S1. Primers of amplifying and sequencing primers for three SNPs.**

| **Primer name** | **Sequences** | **Production length** |
| --- | --- | --- |
| rs77076061-F | CCGCCGCCACCATGTCTCAGGA | 458 bp |
| rs77076061-R | CCCCCATCTCTAGGCACCGTTCTC |  |
| rs1979262-F | CAGGCATTGTCGGTCCATAACTC | 627 bp |
| rs1979262-R | CGCCTCGGCCTCCCAAAAT |  |
| rs12611087-F | TGAGGTCAGGAGTTCGAGATTAGC | 454 bp |
| rs12611087-R | TCCAGGTGGCCAGGGTTCAAA |  |
