# Supplementary material for: Association of genetic polymorphisms in the C19orf66 gene and biochemical indices of HBV infected individuals in Yunnan
Source: Front Cell Infect Microbiol. 2023 May 24;13:1180366. doi: 10.3389/fcimb.2023.1180366 (PMC10245551; doi:10.3389/fcimb.2023.1180366)
Supplement: Supplementary file 2 [file Table_2.docx]

**Table S2. Primers of amplifying and sequencing primers for three SNPs.**

| **Primer name** | **Sequences** | **restriction endonuclease site** | **Production length** |
| --- | --- | --- | --- |
| rs77076061-pGL3-Basic-F | GGTACCAGGCCAGAGATAACCTGG | *Kpn* I | 178 bp |
| rs77076061-pGL3-Basic-R | GTTCGAAAACTTGACCCTTGGTTTTGG | *Hind* III |  |
| rs1979262-pGL3-Basic-F | GGGTACCGGAAAGAAAGGCAGGGGTCTT | *Kpn* I | 171 bp |
| rs1979262-pGL3-Basic-R | GTTCGAATCATTCATTCATTCATTCAACACAAG | *Hind* III |  |
| rs12611087-pGL3-Basic-F | GGTACCGGCAGGCTAAGGCAGAAGAATCACT | *Kpn* I | 194 bp |
| rs12611087-pGL3-Basic-R | GGTTCGAATCAATGAATGCTACTCTCTGCTAAT | *Hind* III |  |
